# *Coelogyne victoria-reginae* (Orchidaceae, Epidendroideae, Arethuseae), a new species from Chin State, Myanmar

**DOI:** 10.3897/phytokeys.98.23298

**Published:** 2018-05-18

**Authors:** Shi-Shun Zhou, Yun-Hong Tan, Xiao-Hua Jin, Kyaw Win Maung, Myint Zyaw, Ren Li, Rui-Chang Quan, Qiang Liu

**Affiliations:** 1 Southeast Asia Biodiversity Research Institute, Chinese Academy of Sciences, Yezin, Nay Pyi Taw 05282, Myanmar; 2 Xishuangbanna Tropical Botanical Garden, Chinese Academy of Sciences, Mengla, Yunnan 666303, China; 3 State Key Laboratory of Systematic and Evolutionary Botany, Institute of Botany, Chinese Academy of Sciences, Beijing 100093, China; 4 HponkanRazi Wildlife Sanctuary Offices, Putao, Myanmar; 5 Forest Research Institute, Forest Department Ministry of Environmental Conservation and Forestry, Yezin, Nay Pyi Taw, Myanmar

**Keywords:** NatmaTaung (Mt. Victoria) National Park, risk of extinction assessment, section *Proliferae*, taxonomy

## Abstract

*Coelogyne victoria-reginae*, a new species of section Proliferae, from Natma Taung (Mt.Victoria) National Park, Chin State, Myanmar, is described and illustrated. It is morphologically similar to *C. prolifera*, but the clustered pseudobulbs, pure brownish- red flowers and column wing with irregular notches at the apex of the new species differ from the other species. A preliminary risk-of-extinction assessment shows that the new species is regarded as EN C2a[i] according to the IUCN Red List Categories and Criteria.

## Introduction

*Coelogyne* Lindley (1821) is characterised by a free, never-saccate lip, with high lateral lobes over the entire length of the hypochile and smooth, papillose, toothed or warty keels on the epichile (Seidenfaden and Wood 1992). This genus comprises over 200 species, distributed from Southeast Asia to the south-western Pacific Islands (Butzin 1992, Clayton 2002, Chen and Clayton 2009). Around 46 species of this genus have been recorded from Myanmar (Kress et al. 2003, Kurzweil and Lwin 2014, Aung et al. 2017).

The Natma Taung (Mt. Victoria) National Park is located in the south-western part of Myanmar. Mount Victoria is the highest mountain in this range and regarded as an ecological refugium, offering a temperate climate that is absent from neighbouring regions (Tanaka et al. 2010a). It is estimated that there are about 2500 vascular plant species on Mt. Victoria (Mill 1995) and a number of endemic and relict species have been found in this area (Hutchinson 1917, Cowley 1982, Ikeda and Ohba 1995, Tanaka et al. 2010a, 2010b, Yukawa et al. 2010). During our field expeditions in this area since 2016, carried out by the Xishuangbanna Tropical Botanical Garden, CAS, in cooperation with the Forest Department, Union of Myanmar Ministry of Forestry, a new species of *Coelogyne* was discovered and is described below. The new species belongs to Coelogyne section Proliferae (Lindl.) Pfitzer and Kraenzlin (1907).

## Materials and method

Morphological descriptions (Stearn 1983) of the new species were carried out based on five living plants and three dried herbarium specimens (HITBC: herbaria of Xishuangbanna Tropical Botanical Garden, the Chinese Academy of Science). Measurements were made using a vernier caliper. Herbarium or fresh specimens of *Coelogyne prolifera* (PE: herbaria of Institute of Botany, the Chinese Academy of Science), *C. schultesii* (CAL: herbaria of Calcuttense), *C. ustulata* (K: herbaria of Royal Botanical Garden, Kew) and *C. ecarinata* (HTIBC) were also examined. The conservation status of the new species was evaluated based on the International Union for Conservation of Nature standard of the criteria C (Small population size and decline): C1 is an observed, estimated or projected continuing decline of at least up to a maximum of 100 years in the future; C2 is an observed, estimated, projected or inferred continuing decline and including at least 1 of the following 3 conditions: a [i] number of mature individuals in each subpopulation; a [ii] percentage (%) of mature individuals in one subpopulation; (b) extreme fluctuations in the number of mature individuals. Here, we have just observed the number of mature individuals in the subpopulation and criteria of C2a [i]is used to evaluate the threatened status, of which the number of mature individual ≤ 50 is CR (critically endangered); ≤ 250 is EN (endangered); ≤ 1000 is VU (vulnerable) (IUCN Standards and Petitions Subcommittee 2017).

## Taxonomic treatment

### Coelogyne victoria-reginae

Taxon classificationPlantaeAsparagalesOrchidaceae

Q.Liu & S.S.Zhou sp. nov.

urn:lsid:ipni.org:names:77178924-1

Figs 1 , 2

#### Diagnosis.

*Coelogyne victoria-reginae* is closely related to *C. prolifera* by having the elliptic mid-lobe with two lamellae terminating at 2/3 on to mid-lobe, ovate or oblong lateral lobes. However, the new species can be distinguished from the latter by the ovoid pseudobulb and 1.1–1.4 cm apart on rhizome, flower brownish-red, lateral sepals (10–11 ×5.5–6.0 mm) significantly larger than dorsal sepal (7.0–8.0 × 4.5–5.0 mm).

#### Type.

MYANMAR. Chin State. Natma Taung (Mt. Victoria) National Park, subtropical evergreen broad-leaved forest, 2400–2600 m, epiphytic on the upper trunk, 1 May 2017, Qiang Liu, *M17-18* (holotype, HITBC!, isotypes, RAF!).

#### Description.

Epiphytic, rhizome creeping, rigid, 3.5–4.5 mm in diameter, densely covered with leathery scaly sheaths, with rather short internodes. Pseudobulbs 1.1–1.4 cm apart on rhizome, globose or ovoid-oblong 3.1–4.0 × 1.0–1.5 cm. Leaves two on each pseudobulb, terminal, oblong lanceolate, coriaceous, 6.5–8.3 × 2.4–3.1 cm, apex acuminate; petiole 1.0–1.7 cm. Inflorescence arising from top of mature pseudobulbs, up to 21.5–32.3 cm long, 4–6-flowered, far longer than leaves, with many persistent distichous sterile bracts just below rachis and several closely spaced distichous sterile bracts at apex of the rachis. Rachis extending and producing annual sets of imbricate bracts and flowers. Floral bracts lanceolate, almost deciduous at anthesis, ca. 1.2 cm; pedicel and ovary 8.0–10.0 mm. Flowers brownish-red, dorsal sepal triangular-ovate, 7.0–8.0 × 4.5–5.0 mm, acuminate; lateral sepals similar to dorsal sepal,10.0–11.0 × 5.5–6.0 mm. Petals narrowly linear, acuminate, 9.0–10.0 × 1.0 mm; lip 3-lobed, subovate, 10.0–11.0 × 7.0–8.0 mm, callus with 2 conspicuous longitudinal lamellae extending from base of mid-lobe; lateral lobes erect, ovate, 5.0 × 3.0 mm; mid-lobe nearly elliptic, reflexed, ca. 6.0 × 5.0 mm, margin undulate, apex emarginate; column ca. 6.0 mm, apex winged with serration; anther cap coniform; pollinia four, semi-orbicular.

#### Etymology.

The new species is named after Victoria Mountain region, NatmaTaung National Park, Chin State, South-western Myanmar, where it was discovered in a vast area of mountain forest.

#### Phenology.

Flowering occurs in April and May.

#### Distribution and habitat.

*Coelogyne victoria-reginae* is only known from the type locality. It grows as an epiphyte on tree trunks in subtropical evergreen broad-leaved forest, which is dominated by *Lithocarpus xylocarpus* (Kurz) Markg. (Fagaceae).

#### Conservation status.

*Coelogyne victoria-reginae* was collected in the Victoria Mountain, Natma Taung National Park, Chin State, South-western Myanmar. However, only one population, consisting of approximately100 individuals, has been discovered so far in the National Park. Although other populations may be found with further investigation because the area is legally protected under the management of the Myanmar Forest Department, the number of mature individuals of the subpopulation may be less than 250 on account of similar habitat. Thus, the species is here assigned a preliminary status of EN C2a [i]according to the guidelines for using the IUCN Red List Categories and Criteria (IUCN Standards and Petitions Subcommittee 2017).

##### Key to Coelogyne sect. Proliferae

**Table:** 

1	Lip without lamellae, lateral lobes semi-orbicular in size	**2**
–	Lip with 2 lamellae, lateral lobes ovate or oblong	**3**
2	Flower brownish-red and lip white with a black apex, labellum large (15.0 × 9.0 mm), mid-lobe rotund	***C. ecarinata***
–	Flower pale yellow-green, labellum small (6.5 × 6.0), mid-lobe semi- elliptic	***C. ustulata***
3	Mid-lobe orbicular or sub-quadrate, with 2 lamellae faint near base of mid-lobe	***C. schultesii***
–	Mid-lobe nearly elliptic, with 2 lamellae terminating at 2/3 on to mid-lobe	**4**
4	Pseudobulb ovoid and 1.1–1.4 cm apart on rhizome, flower brownish-red, lateral sepals (10–11 × 5.5–6.0 mm) significantly larger than dorsal sepal (7.0–8.0 × 4.5–5.0 mm)	***C. victoria-reginae***
–	Pseudobulb narrowly ovoid-oblong and 2.5–4.0 cm apart on rhizome, flower green or yellow green, lateral sepals (3.0-3.6×1.2 mm) equal or smaller than dorsal sepal (3.0-4.0 × 3.0 mm) in size	***C. prolifera***

**Figure 1.**
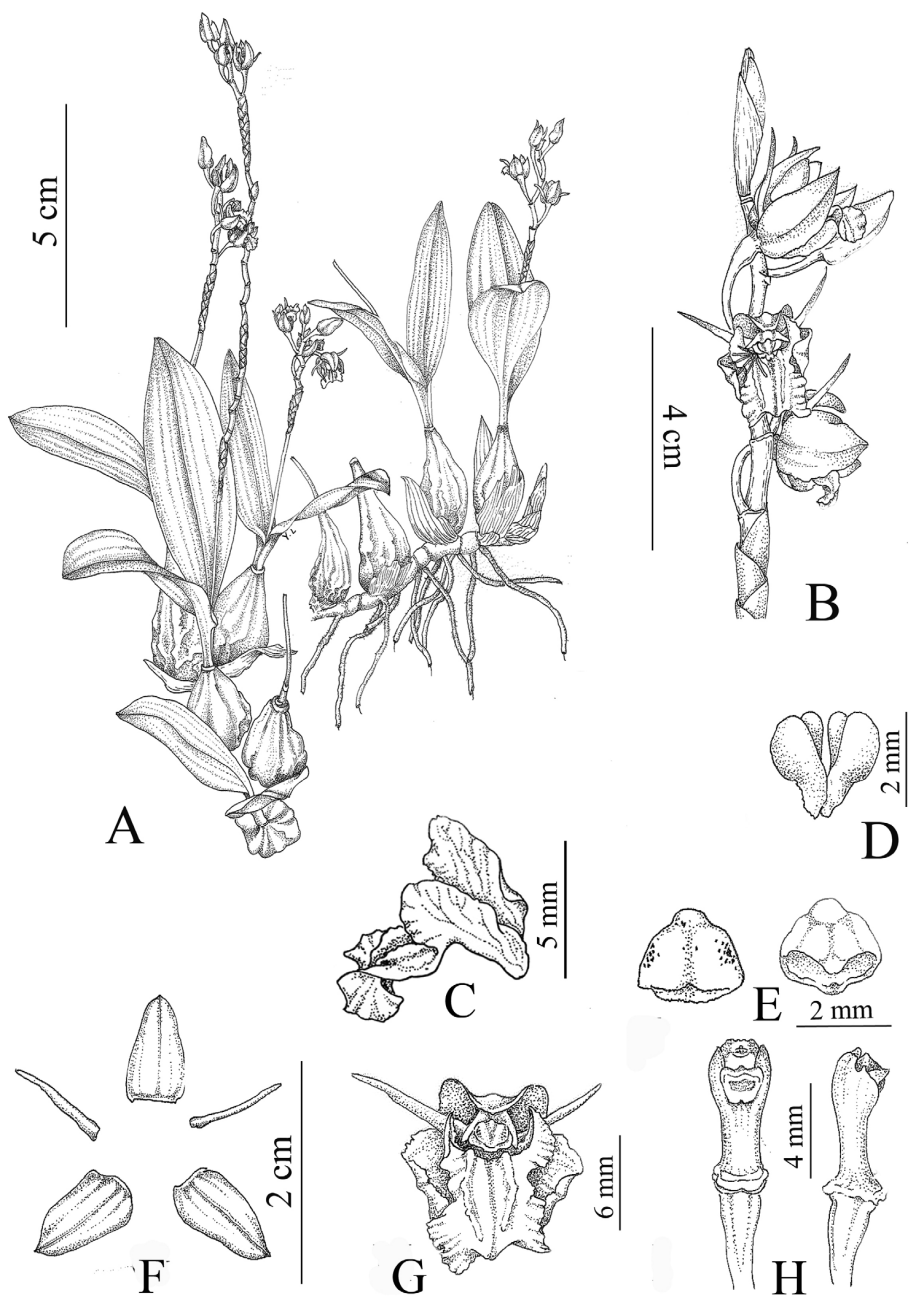
*Coelogyne victoria-reginae*. **A** Plant **B** Inflorescence **C** Lateral view of labellum **D** Pollinarium **E** Abaxial and adaxial anther cap **F** Sepals and petals **G** Front view of flower **H** Front and lateral view of column. All from the type collection (Qiang Liu, *M17-18*) and drawn by Lan Yan.

**Figure 2.**
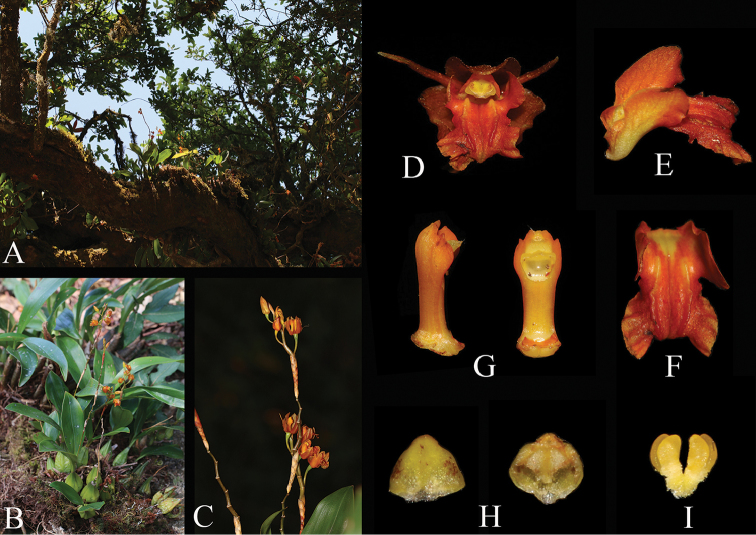
*Coelogyne victoria-reginae*. **A** Habitat **B** Plant **C** Inflorescence **D** Flower **E** Lateral view of labellum **F** Front view of labellum **G** Front and lateral view of column **H** Abaxial and adaxial anther cap. I. Pollinarium (Photographed by Q. Liu)

**Figure 3.**
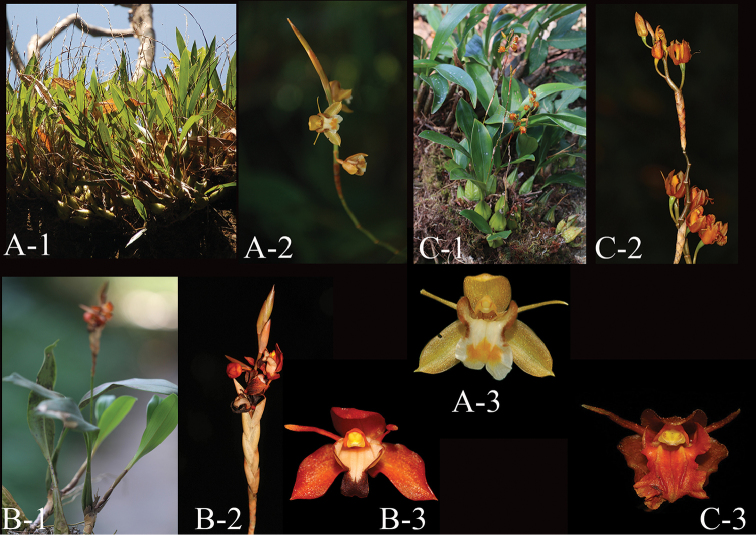
**A** *Coelogyne schultesii* (**A-1** Plant **A-2** Inflorescence **A-3** Flower) **B** *Coelogyne ecarinata* (**B-1** Plant **B-2** Inflorescence **B-3** Flower) **C** *Coelogyne victoria-reginae* (**C-1** Plant **C-2** Inflorescence **C-3** Flower) (Photographed by Q. Liu).

## Discussion

*Coelogyne* was established by Lindley in 1821 and is currently divided into 4 subgenera and 19 sections (Gravendeel 2005). Although revisions of several sections have been published in the last decade (Gravendeel and de Vogel 1999, Pelser et al. 2000), a comprehensive infrageneric delimitation combined with descriptions of morphological characters and molecular phylogeny within *Coelogyne* is needed (Gravendeel 2000, Sierra et al. 2000). Morphologically, the inflorescence of this new species with imbricate sterile bracts at the junction of the peduncle and rachis indicate that it belongs to the section Proliferae (Pfitzer and Kraenzlin 1907, Seidenfaden 1975, Chen et al. 1999). *Coelogyne victoria-reginae* is similar to *C. prolifera*, *C. ustulata*, *C. schultesii* and *C. ecarinata*, both in vegetative morphology and shape of the flowers. However, the new species differs from *C. ustulata* by its 2 longitudinal lamellae on the mid-lobe and lateral lobes smaller than the mid-lobe (mid-lobe without 2 longitudinal lamellae and lateral lobes significantly larger than mid-lobe in *C. ustulata*) (Seidenfaden 1975, Pedersen et al. 2014). It differs from *C. ecarinata* by its small and tight sterile bracts at the apex of the rachis and a brownish- red lip without a black tip (large and loose sterile bracts at apex of rachis and white lip with black tip in *C. ecarinata*) (Jin and Li 2006, Kurzweil and Lwin 2014). It differs from *C. schultesii* by its brownish-red flowers, lateral sepals (10.0–11.0× 5.5–6.0 mm) larger than dorsal sepal (7.0–8.0 × 4.5–5.0 mm), ovate lateral lobes and size is 5.0 × 3.0 mm, elliptic mid-lobe and size is 6.0 × 5.0 mm, apex of column wing is significantly notched (brownish-yellow to dark brown sometimes light greenish flowers, lateral sepals (12.0-18.0× 4.0-6.0 mm) as large as dorsal sepal (12.0–18.0× 6.0–9.0 mm), oblong lateral lobes and size is 8.0–12.0× 3.0–5.0 mm, orbicular or subquadrate mid-lobe and size is 7.0–10.0 × 8.0–11.0 mm, apex of column wing is entire in *C. schultesii*) (Seidenfaden 1975, Jain and Das 1978, Chen and Clayton 2009). It differs from *C. prolifera* by having globose pseudobulbs, 1.1–1.4 cm apart on the rhizome and conical anther cap (narrowly ovoid-oblong pseudobulb, 2.5–4.0 cm apart on rhizome and subglobose anther cap in *C. prolifera*) (Seidenfaden 1975, Chen and Clayton 2009). Meanwhile, the new species is only growing in the subtropical evergreen broad-leaved forest at 2400–2600 m in South-western Myanmar; *Coelogyne prolifera* is growing in the montane rain forest or subtropical evergreen broad-leaved forest at 1100–2200 m in Southeast Asia; *C. ustulata* is growing in the montane rain forest at 1700–1800 m in Myanmar and Thailand; *C. schultesii* is growing in the subtropical evergreen broad-leaved forest at 1700–2000 m in Bhutan, India, Myanmar, Nepal, Thailand, Vietnam and China; *C. ecarinata* is growing in the montane rain forest at 1000–2600 m in North of Myanmar and Yunnan of China (Seidenfaden 1975, Jin and Li 2006, Chen and Clayton 2009).

## Supplementary Material

XML Treatment for Coelogyne victoria-reginae
